# Performance Design of Bio-Inspired Arc-Circular Honeycombs Under In-Plane Loading

**DOI:** 10.3390/biomimetics11010033

**Published:** 2026-01-04

**Authors:** Chengliang Zhu, Yangyang Liu

**Affiliations:** 1 School of Engineering, Anhui Agricultural University, Hefei 230036, China; 2 School of Mechanical Engineering, Anhui University of Technology, Ma’anshan 243032, China

**Keywords:** mechanical property, energy absorption, honeycombs, in-plane loading

## Abstract

This study proposes an arc-circular lightweight honeycomb structure. Three different configurations of honeycomb specimens, namely arched honeycombs (AHs), arc-circular honeycombs with a first-order hierarchical configuration (ACH-1), and arc-circular honeycombs with a second-order hierarchical configuration (ACH-2), are prepared using metal additive manufacturing technology, and quasi-static compression tests are conducted. The results show that all configurations exhibit significant multi-stage load responses, with the ACH-2 configuration, which incorporates smaller sub-cells, demonstrating higher compressive stress and energy absorption potential. The specific energy absorption (*SEA*) of ACH-2 is enhanced by 210% compared to the baseline AH. The effectiveness of the finite element analysis is validated against experimental results. Further parametric analysis of the wall thickness parameters, cell number, and macroscopic dimensions of ACH-2 reveals significant variations in how wall thickness at different local locations affects the mechanical properties. Additionally, although increasing the macroscopic dimension significantly enhances the energy absorption capacity, the effect of increasing the number of cells on the overall energy absorption performance at the same relative density is limited. Finally, a reverse design framework for ACH-2 with multi-stage plateau stress is established. The effectiveness of this performance design framework is validated through experiments, providing a feasible technical approach for the design of honeycomb structures with multi-stage plateau stress characteristics.

## 1. Introduction

Lightweight honeycomb structures have transformed engineering design by delivering exceptional specific strength and superior energy absorption [1,2,3]. Among these benefits, honeycomb structures stand out as particularly promising due to their efficient material usage and predictable deformation behavior under mechanical loads [4]. The key advantage of honeycomb architectures lies in their ability to distribute stresses uniformly throughout their cellular network, allowing them to sustain significant loads while remaining lightweight. This unique combination of properties has made honeycombs indispensable in applications where weight reduction is essential, without sacrificing structural integrity or safety performance. As a result, a variety of high-performance honeycomb designs have been successfully implemented across automotive, aerospace, and marine industries. Current research on honeycomb structures is primarily focused on the development of novel mechanical topologies and customized design strategies tailored to specific mechanical properties. Recent advances in additive manufacturing (AM) have further expanded the design and fabrication potential of honeycomb structures [5,6,7,8,9]. Unlike conventional manufacturing techniques, which impose severe limitations on geometric complexity, AM facilitates the production of intricate cellular architectures with finely tuned mechanical characteristics. This manufacturing flexibility enables engineers to explore bio-inspired designs, emulating optimization strategies developed through millions of years of evolution in natural structures [10]. Consequently, the integration of AM with honeycomb design has opened new opportunities in the development of lightweight structures with enhanced performance [11,12].

In recent years, some high-performance honeycomb structures have gradually been designed to study their excellent mechanical properties [13,14,15,16]. For example, Zhai et al. [17] proposed a pre-folded honeycomb, an energy-absorbing structure created by adding pre-folded traces to conventional honeycombs, and investigated its folding behavior and energy absorption performance under in-plane impact through numerical and experimental methods. Their results showed that the in-plane strength of the pre-folded honeycomb is nearly eight times that of conventional honeycomb. Li et al. [18] developed a new theoretical approach to characterize the in-plane yield strength anisotropy of square honeycombs. They identified four typical deformation modes, including a unique rotating mechanism under small-angle off-axis compression. These findings provide important insights for tailoring the off-axis mechanical performance of honeycombs in engineering applications. Additionally, Lu et al. [19] proposed both forward analytical and inverse numerical approaches to determine the equivalent elastic constants and loss factors of spiderweb-inspired hierarchical composite honeycombs. Specifically, a mechanical model based on beam and plate theory was developed for forward parameter calculation, complemented by an inverse identification procedure using modal data from simulations and experiments. Together, these approaches offer a reliable reference for identifying material parameters of complex hierarchical sandwich honeycombs. Wei et al. [20] proposed a novel star-shaped re-entrant circular (SRC) honeycomb, designed by embedding circular structures into a star-shaped framework with shortened vertical ligaments. This design enhances the compressive stiffness of auxetic materials while preserving their negative Poisson’s ratio. The SRC honeycomb exhibits an “I”-shaped deformation mode, leading to significantly improved compressive stiffness and specific energy absorption. Qiao et al. [21] introduced an auxetic columnar honeycomb structure for enhanced crashworthiness, investigating its mechanical properties and energy absorption. By varying the number of unit cells, they analyzed how geometric parameters influence deformation mechanisms and impact resistance, revealing a unique compression–torsion coupled deformation mode under impact. Omar El Khatib et al. [22] developed an analytical model to predict the in-plane elastic–plastic response and collapse stress of hierarchical honeycombs with sandwich-structured cell walls (SSHCs) under multiaxial loading. Peng et al. [23] employed a two-dimensional equivalent homogeneous model based on the variational asymptotic method to analyze the equivalent elastic properties and impact stresses of four auxetic honeycombs. Yu et al. [24] designed and fabricated a novel bamboo honeycomb material using bamboo integrated panels and hexagonal cores, evaluating its in-plane compression performance through analytical, numerical, and experimental methods. To enhance the impact of energy absorption and plateau stress uniformity, Zhang et al. [25] proposed a bio-inspired re-entrant arc-shaped honeycomb (RAH). Their study demonstrated that the RAH offers superior crushing stability compared to conventional re-entrant honeycombs, with dynamic behaviors governed by relative density, impact velocity, and curvature-based cell parameters. Based on a hierarchical design strategy, Ma et al. [26] developed a vertex-based hierarchical re-entrant origami honeycomb (VHROH) to improve crashworthiness. Parametric studies confirmed that crashworthiness can be effectively regulated by adjusting substructure size and folding angle, albeit at the expense of a weakened negative Poisson’s ratio effect. The studies reviewed above demonstrate that the mechanical properties of lightweight honeycomb structures, such as strength, stiffness, and energy absorption capacity, can be effectively tailored through modifications in their internal geometry (i.e., topological form). This advancement has moved honeycomb design beyond traditional single-cell configurations, enabling the creation of structures that optimally combine low weight with specific mechanical performance. However, while significant progress has been made in maximizing overall energy absorption efficiency, modern engineering applications increasingly demand adaptive protection systems capable of responding to a wide range of impact energies. This necessitates the development of structures exhibiting a multi-stage load response, which is characterized by multiple distinct stress plateaus. Such a response, achieved by controlling sequential buckling or other transient deformation mechanisms, provides superior buffering and energy management across varying loading conditions.

To improve the above issues, several innovative designs featuring multi-stage responses have been developed. Ding et al. [27] proposed a bio-inspired locally enhanced re-entrant honeycomb (LERH) using hierarchical and hybrid design, integrating parallelogram and triangular structures. Their quasi-static compression tests and numerical simulations revealed a unique two-stage plateau behavior, with the second plateau stress 242.59% higher than the first. Similarly, Li et al. [28] developed a rotating star-rhombic auxetic structure (RSAS) based on rigid rotating units, which exhibits a significant negative Poisson’s ratio and enhanced energy absorption. Their experiments and simulations identified a tunable multi-stage deformation mode, where the second plateau stress varies predictably with geometric parameters such as re-entrant angle and wall thickness. In pursuit of higher energy absorption, Wang et al. [29] integrated double arrowhead cells into a star-shaped honeycomb to create a star-arrowhead honeycomb (SAH). This structure exhibits two distinct plateau stress regions under low-velocity impact, with the second exceeding the first by over three times. Notably, the SAH uniquely transitions from a negative to a positive Poisson’s ratio under low- and medium-velocity impacts, a behavior not observed in conventional counterparts. Further exploiting hierarchical design, Lu et al. [30] introduced a rotating star-circle structure (RSCS) by modifying a classical rotational system with beam-like links and a star-circle substructure. The RSCS demonstrates a distinctive three-step deformation mode with three corresponding plateau stages and exhibits superior specific energy absorption compared to other auxetic structures. Chen et al. [31] approached the challenge through self-similar nesting, proposing SN-SC, SN-FCC, and SN-BCC units. Their analysis revealed that the dual-plateau mechanism originates from the division of each unit into a stronger inner region (containing the nested core) and a weaker outer region, with the core generating the elevated second stress plateau. In an applied engineering context, Zhang et al. [32] designed a negative Poisson’s ratio (NPR) block as a bridge girder falling restrainer. Monotonic loading tests and simulations confirmed its three-stage deformation process, where both stiffness and plateau load increase significantly after the initial phase, fulfilling requirements for high energy absorption and bearing capacity. Collectively, these studies demonstrate significant progress, and the underlying principles and potential of multi-stage energy absorption in honeycomb structures merit continued and deeper exploration.

Inspired by biomimetic hierarchical design and the properties of higher-order arc-circular honeycombs, this study investigates their multi-stage mechanical behavior. The remainder of this paper is organized as follows. Section 2 details the design concept, fabrication process, and experimental methodology of the arc-circular honeycomb. Section 3 examines its key mechanical parameter effects. Section 4 discusses the potential for customizing its multi-stage mechanical response. Finally, Section 5 summarizes the main conclusions of this work.

## 2. Design and Experiments

Biomaterials, representing nature’s evolved ingenuity, establish a key paradigm for structural biomimicry aimed at overcoming performance limitations in engineering materials [33,34]. Through effective multi-scale structural design, they achieve synergistic combinations of mechanical properties, including high strength, toughness, and fatigue resistance, which are difficult to attain using a single homogeneous material. This success charts an innovative course for the integrated design of structure, function, and performance in engineered materials. Notably, the hierarchical architecture intrinsic to many biomaterials holds particular significance for the mechanical design of lightweight honeycomb structures [35,36,37]. By emulating these biological hierarchical features, the mechanical properties of such honeycombs can be substantially enhanced. Crucially, this improvement in impact resistance and energy absorption efficiency can be achieved while retaining low density, thereby pioneering new directions for biomimetic design in weight-critical fields, including aerospace and automotive engineering.

**Figure 1:**
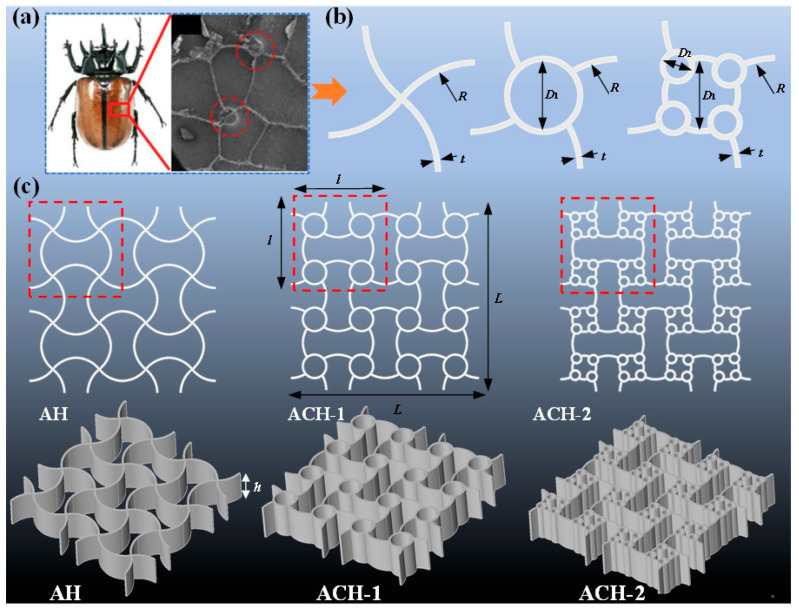
Design concept of arc-circular honeycombs: (**a**) Cross-sectional internal structures of the beetle elytra [38]; (**b**) design method of arc-circular honeycombs; (**c**) arched honeycombs (AH), arc-circular honeycombs with a first-order hierarchical configuration (ACH-1), and arc-circular honeycombs with a second-order hierarchical configuration (ACH-2).

The columnar structures within beetle elytra have attracted particular research interest due to their distinctive and mechanically promising bioinspired characteristics, as illustrated in Figure 1a [14,38]. Inspired by this model, the present work introduces bio-inspired hollow connective cells into arched honeycomb structures, effectively replacing the original nodal connections (Figure 1b). This substitution process is iterated to incorporate additional circular cells, thereby progressively increasing the structural complexity. The generated cells are then systematically assembled into a complete, functional honeycomb through mirroring and array operations. This core design strategy, which replaces original cross-sectional connections with smaller constituent cells, is supported by prior literature demonstrating its efficacy in achieving enhanced performance [19,26,39,40]. Applying this method, three distinct honeycomb structures are designed, as shown in Figure 1c: arched honeycombs (AH), arc-circular honeycombs with a first-order hierarchical configuration (ACH-1), and arc-circular honeycombs with a second-order hierarchical configuration (ACH-2). The design concepts of AH and ACH-1 have also been mentioned in existing literature [41]. All three variants are derived from a common design framework and consequently share identical macroscopic dimensions.

**Figure 2:**
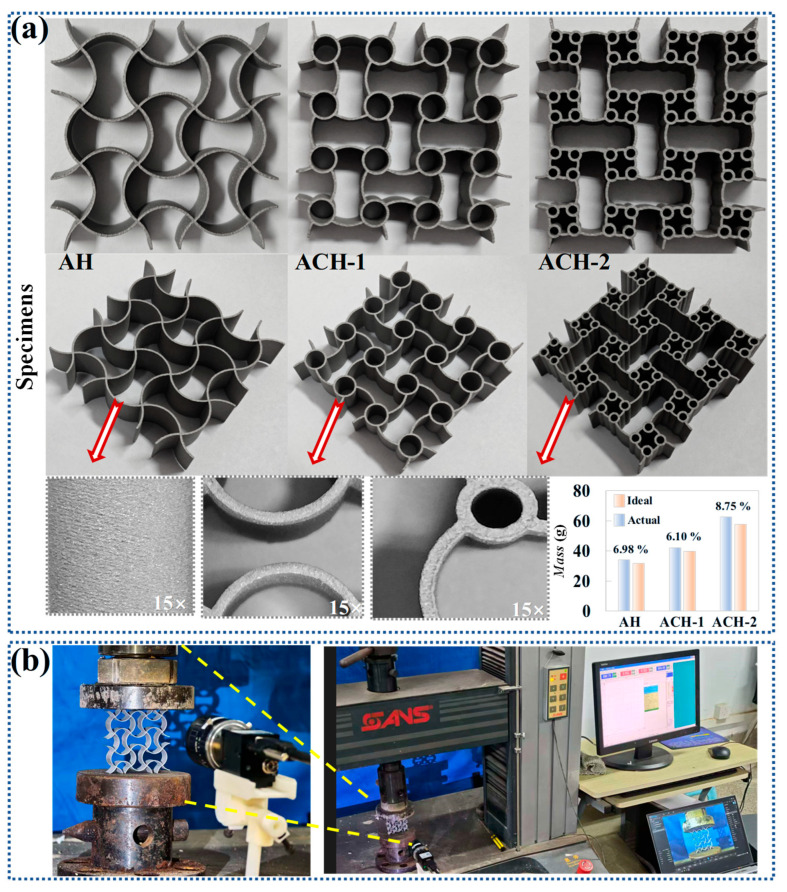
Experiment on arc-circular honeycombs: (**a**) specimens printed through additive manufacturing; (**b**) experiment process.

Laser Powder Bed Fusion (LPBF) is an additive manufacturing technique that selectively melts metal powders with a laser, building three-dimensional parts layer by layer. It is known for its high precision and capability to produce complex geometries. In this study, honeycomb structures are fabricated using LPBF. The key process parameters include support speed, scanning speed, support power, and filling power. Specifically, the support speed is set at 2 mm/min, and the scanning speed at 1800 mm/min. These parameters together control the laser’s energy input and the cooling rate of the melt pool, which in turn affect the quality of the melt tracks and the microstructure of the parts. The support power is set at 47 W, primarily for melting the support structures to ensure process stability. The filling power, set at 62 W, directly influences the melting of the solid part and plays a crucial role in determining the density and mechanical properties of the final part. The laser used in this study has a maximum power of 500 W, providing the necessary energy for powder melting and metallurgical bonding. The specific settings for support and filling power ensure stable melt pool formation and precise process control. As shown in Figure 2a, the AH (*t* = 0.6 mm, *l* = 30 mm, *R* = 10 mm), ACH-1 (*t* = 0.6 mm, *l* = 30 mm, *R* = 10 mm, *D*_1_ = 7.5 mm), and ACH-2 (*t* = 0.6 mm, *l* = 30 mm, *R* = 10 mm, *D*_1_ = 7.5 mm, *D*_2_ = 3 mm) specimens are fabricated as 2 × 2 arrays. Process defects in metal 3D printing can significantly affect the performance of the final printed part. For example, during the cooling phase, uneven cooling rates across different sections of the metal material result in non-uniform thermal expansion, which generates uneven internal stresses. These residual stresses often cause deformation or warping of the workpiece. In severe cases, they may compromise its geometric accuracy and further degrade the mechanical properties of the printed part. Additionally, higher surface roughness tends to reduce the fracture toughness of metal 3D-printed components. A rough surface can easily become a source of crack initiation. Under external loading, such cracks can propagate rapidly, leading to a decrease in material toughness. Upon local magnification of the prepared specimens, it can be observed that the surfaces of all metal-printed parts are relatively smooth, flat, and have clear contours. In addition, the maximum error between the ideal weight and the actual weight is within 9%, further indicating that metal printed specimens can be used for experiments.

In this study, a total of 3 specimens are subjected to compression testing, as shown in Figure 2b. All honeycomb cells are placed on a universal material testing machine (CMT5105 electronic universal testing machine, SASTest, Shenzhen, China) for testing. The bottom of the honeycomb contacts a fixed panel, while the top of the honeycomb contacts a movable panel. The experimental compressive speed is set to 2 mm/min. The basic mechanical properties are as follows: material density of 7.86 g/cm^3^, Poisson’s ratio of 0.3, elastic modulus of 186 GPa, and yield stress of 480 MPa. The finite element numerical model of the honeycombs is developed using the nonlinear finite element software LS-DYNA (R11.1). The honeycomb structures are modeled with a piecewise linear elastic–plastic material. The model is discretized using Belytschko–Tsay four-node shell elements, with five integration points in the thickness direction and one integration point in the plane direction. Additionally, the static and dynamic friction coefficients for contact during deformation are set to 0.3 and 0.2, respectively. Numerical simulations of honeycombs with different wall thicknesses using shell elements have been implemented in many studies [42,43], and to balance accuracy and computational cost, convergence analysis is conducted to obtain a shell element size of 0.5 mm. Under low-speed compression, the effect of strain rate on mechanical properties is relatively small. Quasi-static process still can be achieved with high artificial velocity, provided that the inertial effect is minimized. To reduce computational cost, the compressive speed of simulation models is set to 1 m/s [44,45].

**Figure 3:**
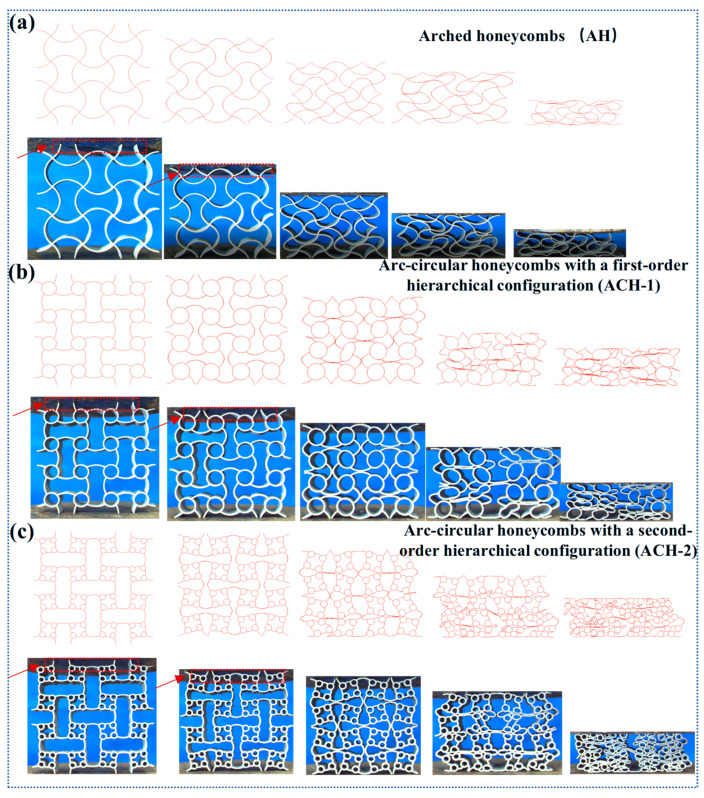
Deformation modes of arc-circular honeycombs: (**a**) AH; (**b**) ACH-1; (**c**) ACH-2.

**Figure 4:**
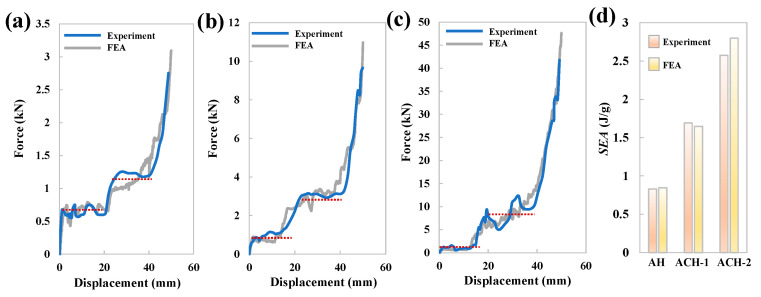
Results of experiment and FEA: (**a**) crushing forces of AH; (**b**) crushing forces of ACH-1; (**c**) crushing forces of ACH-2; (**d**) specific energy absorption.

The investigation began by analyzing the mechanical response and deformation characteristics of the three honeycomb structures. Figure 3 illustrates the observed deformation modes, while Figure 4 presents the corresponding compression load curves. Notably, the experimental and finite element analysis (FEA) results showed a high degree of consistency across all measured aspects, providing initial validation for the numerical approach. Specifically, the deformation modes shown in Figure 3 indicate that, during the compression process, the arched wall is the first part to undergo torsional deformation, followed by the compression of cells with smaller cross-sectional dimensions. In terms of the mechanical response, the load curves in Figure 4 consistently exhibit two-stage plateau stress for all three structures. This analysis confirmed that the maximum error between the two datasets is less than 8.5%, validating the reliability of the numerical model used in this study. *SEA* represents the energy absorbed per unit mass, which is a key indicator for determining the absorption capability, and can be calculated as [46,47,48,49,50]

(1) $$SEA=\frac{EA}{Mass}$$

where *Mass* denotes the total weight of honeycombs, *EA* represents the total energy absorption and can be calculated as

(2) $$EA=\int_{0}^{D_{s}}F(x)dx$$

where F(x) denotes the instantaneous compression force, $D_{s}$ is the compression displacement. Here, $D_{s}$ is determined with reference to 60% of the compression direction height of the honeycomb. As shown in Figure 4d, the energy absorption capacity of the three structures is assessed: ACH-2 exhibited the highest performance, followed by ACH-1, with AH demonstrating the lowest capacity. Most notably, the specific energy absorption (*SEA*) of the ACH-2 structure is enhanced by 210% compared to the baseline AH structure. This significant improvement strongly demonstrates that the proposed strategy of adding new cells at the connection angles of the arched honeycomb is highly effective in significantly enhancing its overall energy absorption performance.

## 3. Analysis and Discussion

**Figure 5:**
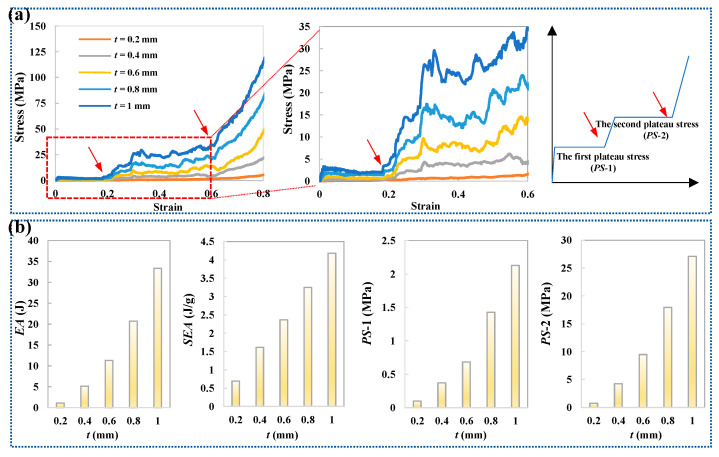
Mechanical properties of honeycombs with different thicknesses: (**a**) crushing forces; (**b**) crashworthiness performances.

**Figure 6:**
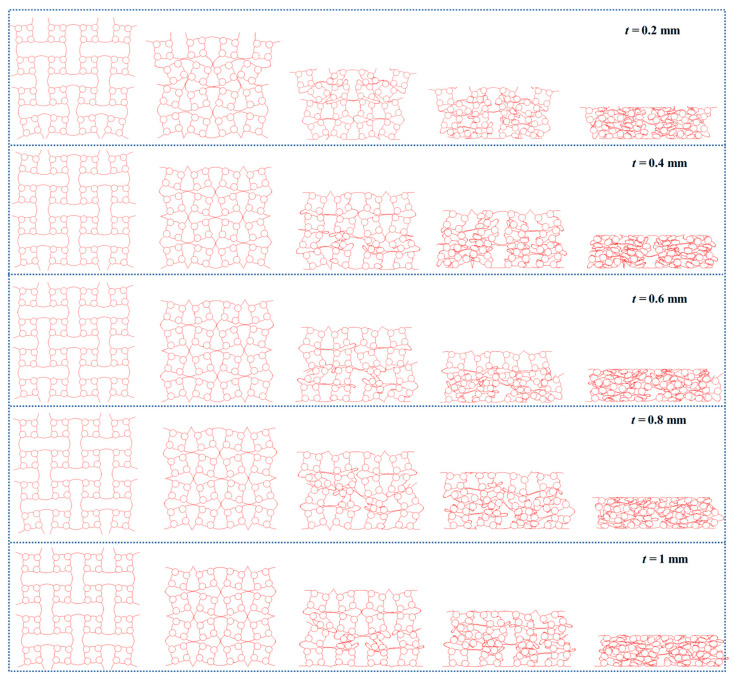
Deformation modes of honeycombs.

Given the superior mechanical properties exhibited by the ACH-2 structure, this study focuses on a detailed parameter analysis, specifically investigating the effect of wall thickness (*t*). To improve computational efficiency, the out-of-plane height of all honeycombs is set to *h* = 1 mm. As shown in Figure 5a, the stress–strain curves under varying wall thickness parameters are presented. It is clearly observed that increasing the wall thickness significantly enhances the overall compressive stress level. Furthermore, all compressive stress curves distinctly exhibit two stable stages of plateau stress (*PS*-1 and *PS*-2). The transition strain between these two-stage plateau stress occurs at approximately 0.2, and the structure enters the densification strain around 0.6. As shown in Figure 6, the corresponding deformation mode analysis reveals the underlying mechanism behind these stages: the first plateau stage is primarily governed by the deformation of the arched wall, while the second plateau stage results from the compression of the circular cells. The synergistic action of these two distinct deformation mechanisms contributes to the continuous plateau stress observed. The transition from the first to the second plateau stress is intrinsic to a shift in the dominant deformation mode. The first plateau stress is primarily governed by the rotational bending of arched walls; when this bending is mechanically suppressed, the stress response undergoes a sequential transition to the second plateau stress, during which all cells deform mainly through compression. Additionally, it is important to note that when the wall thickness is small, the resulting low relative density leads to insufficient overall structural stiffness. As a result, these structures become more susceptible to instability and non-stationary deformation modes during the early stages of compression.

Based on the quantitative results presented in Figure 5b, both the energy absorption capacity and plateau stress levels increase proportionally with the wall thickness parameter. Within the studied parameter range (*t* = 0.2 to 1.0 mm), the total energy absorption showed a significant increase of 29%. Notably, this improvement is 5% higher than the increase observed in the relative density of the structure over the same range, indicating a positive structural efficiency effect beyond simple material addition. Furthermore, the effect of increasing wall thickness on the two-stage plateau stress is analyzed. The stress levels of the first and second plateaus increased by 21% and 36%, respectively. This differential enhancement clearly demonstrates that increasing the wall thickness is significantly more effective in improving the load-bearing capacity associated with the second deformation plateau.

**Figure 7:**
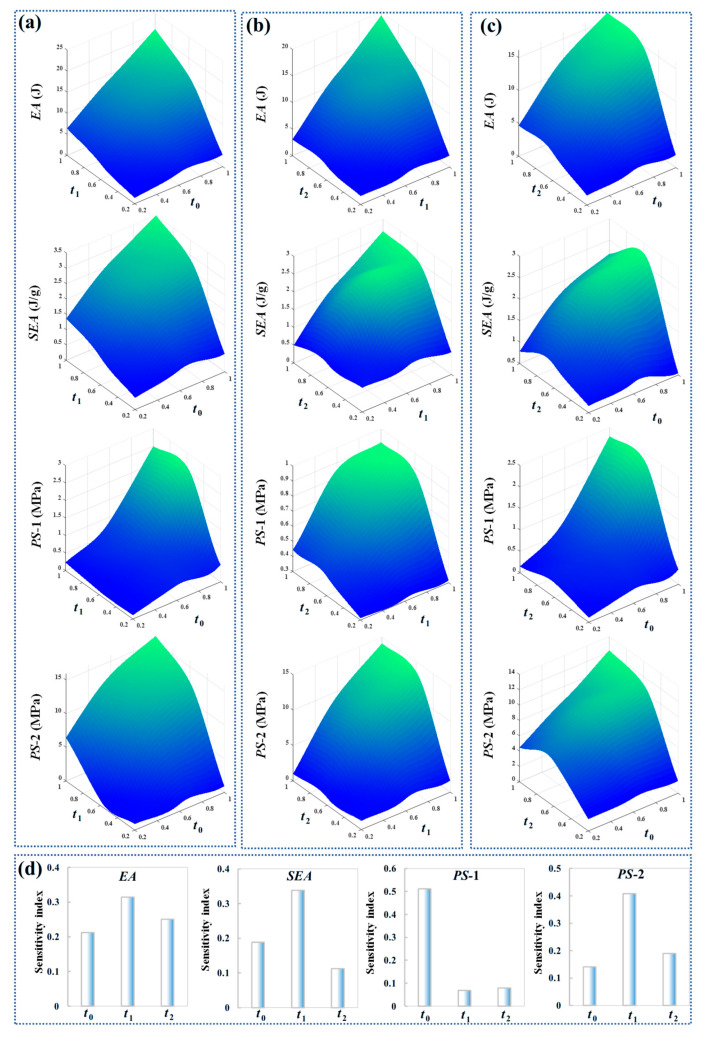
Crashworthiness performances under different local thickness parameters: (**a**) *t*_1_ vs. *t*_0_; (**b**) *t*_2_ vs. *t*_1_; (**c**) *t*_2_ vs. *t*_0_; (**d**) sensitivity index.

This study further investigated the influence of localized design parameters, specifically the wall thicknesses of different structural regions (Arched and circular cells), on the overall mechanical performance. As illustrated in Figure 7, structural properties can be effectively controlled by adjusting the arc wall thickness *t*_0_, the wall thickness *t*_1_ of the first-order circular cell, and the wall thickness *t*_2_ of the second-level circular cell. Energy absorption, specific energy absorption, and plateau stress exhibit a significant nonlinear relationship with these thickness parameters. For example, when *t*_2_ remains constant and *t*_1_ is small, as *t*_0_ increases, energy absorption, specific energy absorption, and plateau stress initially increase and then decrease. Similarly, when *t*_1_ remains constant and *t*_0_ is small, as *t*_2_ increases, energy absorption, specific energy absorption, and plateau stress also follow a trend of first increasing and then decreasing. More specifically, the synergistic increase in both *t*_1_ and *t*_0_ simultaneously enhances multiple performance metrics. This combined adjustment results in improvements in total energy absorption, specific energy absorption, and plateau stress levels for both the first and second deformation plateaus. Therefore, when designing lightweight structures with excellent mechanical performance, the collaborative design of *t*_1_ and *t*_0_ is more effective in controlling the mechanical performance of ACH-2. The sensitivity index obtained using the Sobol method is shown in Figure 7d. It can be observed that *t*_1_ exhibits the highest sensitivity in *EA*, *SEA*, and *PS*-2, and is the main influencing factor of these output parameters. *t*_0_ has the greatest impact on *PS*-1, mainly because the initial triggering deformation mainly occurs on the arched wall. Although increasing design parameters at different locations typically enhances the mechanical performance of a structure, it also entails greater material usage and cost, which contradicts lightweighting objectives. Given that different parameters occupy varying volumes within the structure, their optimal combination must be defined based on core goals, such as weight reduction, strength, or cost.

**Figure 8:**
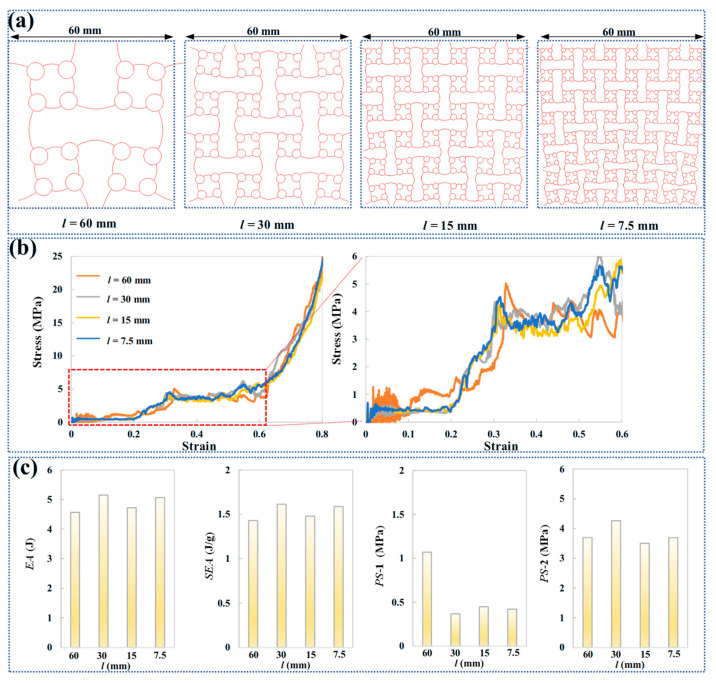
Mechanical analysis of honeycombs with different cell numbers: (**a**) deformation modes; (**b**) compressive stress curves; (**c**) energy absorption, specific energy absorption, and the plateau stress.

The influence of cell numbers on the mechanical properties of ACH-2 is investigated while maintaining a fixed relative density (0.11). Figure 8a illustrates the deformation modes of ACH-2 structures with fixed macroscopic dimensions (60 mm × 60 mm). All ACH-2 configurations exhibit consistent sequential compression deformation modes, starting with the collapse of the arched walls, which constitutes the first stage of plateau stress, followed by the compression of the circular cells, defining the second stage of plateau stress. Figure 8b presents compressive stress corresponding to various compressive strains. It is observed that, despite the addition of more cells, the overall trend of the curve does not significantly change when the relative density is held constant. Moreover, the characteristic strain points remain consistent: the strain corresponding to the end of the first plateau stress stage and the beginning of the second plateau stress stage are approximately 0.2 and 0.3, respectively. The energy absorption, specific energy absorption, and plateau stress metrics all show a nonlinear relationship with the varying cell size. As shown in Figure 8c, when the characteristic cell size *l* varies between 60 mm and 7.5 mm, the rate of change in both energy absorption and specific energy absorption is 13%. In contrast, the change in the first-stage plateau stress is 190%, while the change in the second-stage plateau stress is 22%.

**Figure 9:**
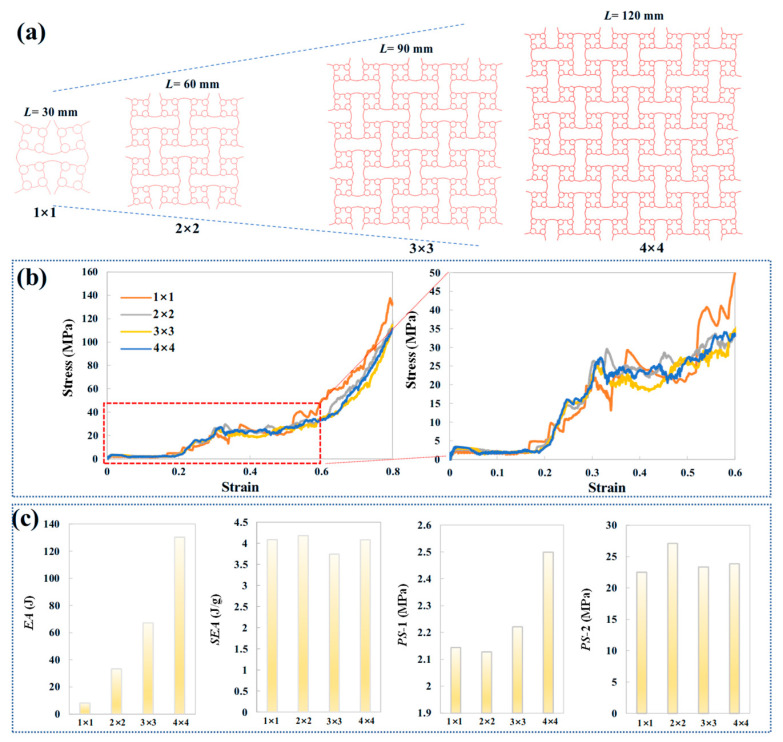
Mechanical analysis of honeycombs with different macroscopic dimensions: (**a**) deformation modes; (**b**) compressive stress curves; and (**c**) energy absorption, specific energy absorption, and plateau stress.

Under the premise of fixing the basic cell size *l* to 30 mm, the influence of macroscopic dimensions on the mechanical properties of ACH-2 is studied. Figure 9a shows the deformation modes of the ACH-2 structures with a fixed basic cell size. All ACH-2 configurations exhibit a consistent continuous compression deformation mode, starting with the collapse of the arched wall, which constitutes the first stage of plateau stress, followed by the compression of the circular cell, which defines the second stage of plateau stress. These phenomena have been described in the previous sections. Figure 9b presents the compressive stress corresponding to various compressive strains. It can be observed that, although the macroscopic dimensions are enlarged, the overall trend of the load curve does not significantly change when the basic cell size remains constant. Additionally, the characteristic strain points remain consistent: the strains corresponding to the end of the first plateau stress stage and the beginning of the second plateau stress stage are approximately 0.2 and 0.3, respectively. Although energy absorption increases with the increase in macroscopic size, there is a nonlinear relationship between specific energy absorption and plateau stress across different macroscopic sizes. As shown in Figure 9c, when the macroscopic dimension *L* varies between 30 mm and 120 mm, the change rate of energy absorption is 15%, while the change rate of specific energy absorption is 12%. The change rate of the first plateau stress is 17%, while the change rate of the second plateau stress is 6%. Increasing the macroscopic size can significantly increase the energy absorption potential, but it does not lead to a substantial increase in energy absorption per unit mass. While increasing the macroscopic size can raise the first plateau stress, the adjustment to the second plateau stress is more limited.

## 4. Performance Design

The reverse design method for structures begins with the target performance and directly generates structural configurations that meet specific mechanical responses using machine learning and topology optimization techniques [51,52,53,54]. Its significance lies in overcoming the limitations of traditional trial-and-error methods, which are reliant on experience, time-consuming, and difficult to manage due to their complex nonlinear relationships. This approach significantly shortens the design cycle, improves efficiency, and opens up possibilities for discovering new functional materials. The core concept involves constructing a data-driven deep neural network model, reverse-deriving structural parameters from known performance requirements, generating high-quality simulation data through finite element analysis to train the model, and using a forward network to verify the rationality of the reverse-generated structure. Ultimately, this leads to rapid mapping from performance to structure, creating new opportunities for the customized application of lightweight mechanical structure design in seismic-resistant buildings, lightweight structures, biomedical implants, and other fields [55].

In this study, this framework first constructs a rich structural design space using parametric methods. Then, a trained machine learning model is employed as an accurate surrogate model to quickly map geometric parameters to macroscopic mechanical properties, bypassing the computational expense of traditional simulations. Global optimization algorithms are used to autonomously explore this design space, identifying the optimal structural configuration that satisfies a specific objective function. The methodology is validated experimentally using advanced technologies such as additive manufacturing, forming a complete closed-loop process from initial performance specifications to final structural generation. Due to the limited range of size ratio variation between small cells in ACH-2, this study primarily focuses on designing different local thickness parameters. The inputs are *t*_0_, *t*_1_, and *t*_2_, and the outputs are *PS*-1 and *PS*-2. Here, 100 sample points are selected using the OLHD method to ensure sufficient coverage of the three-parameter design space [56]. The OLHD method is chosen for its ability to evenly distribute sample points and minimize clustering in low-dimensional parameter spaces. The choice of 100 sample points is based on a balance between computational cost (e.g., finite element simulations for data generation) and the need for sufficient data diversity to train the neural network. To ensure model robustness, a series of evaluations are performed, including train-test split validation and sensitivity analysis of design parameters. A pre-trained neural network (NN) serves as an accurate performance predictor, establishing a rapid mapping relationship between the ACH-2 geometric parameters and macroscopic mechanical responses, replacing computationally expensive finite element simulations. The pre-trained NN is established using a feedforward neural network (FNN) tool in MATLAB (R2020a), which mainly includes two hidden layers. The activation function of the hidden layers is tanh. The first hidden layer has 4 neurons, and the second hidden layer has 3 neurons. The training process uses the Levenberg–Marquardt algorithm, with 20% of the sample points used for validation. The mean square error (*MSE*) and coefficient of determination (*R*^2^) are used as evaluation metrics [57]. The standardized *MSE* and *R*^2^ values for *PS*-1 predictions are 0.08 and 0.92, respectively, while the standardized *MSE* and *R*^2^ values for *PS*-2 predictions are 0.05 and 0.96, respectively.

**Figure 10:**
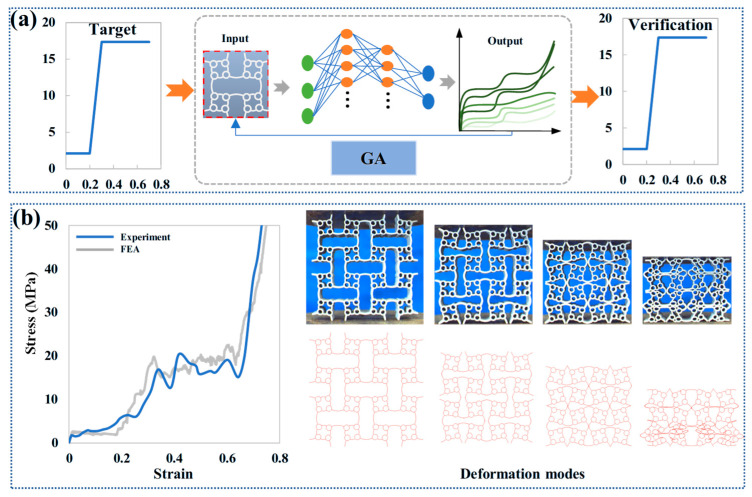
Customized design of multi-stage plateau stress: (**a**) design framework; (**b**) experimental verification.

In this framework, a Genetic Algorithm (GA) acts as the global optimization core, using operations like selection, crossover, and mutation to efficiently explore the ACH-2 design space [58,59]. This method effectively combines the robust search capability of the GA with the highly efficient evaluation provided by the NN, significantly accelerating the design process. The optimization toolbox in MATLAB can implement genetic algorithms, with a population of 20 and a mutation rate of 0.01. As depicted in Figure 10a, the design objective is demonstrated using a pre-specified target plateau stress profile. Assuming the first plateau stress is 2.12 MPa and the second plateau stress is 17.35 MPa, the optimization objective in this study is to minimize the average relative error between the target plateau stress and the predicted plateau stress (seven data points are selected at intervals of 0–0.7 compression strain). The design scheme with the smallest average relative error indicates that the plateau stress of the design is close to the target scheme.

Applying the methodology illustrated in Figure 10a, the optimal design parameters determined for the target performance are *l* = 30 mm, *L* = 60 mm, *t*_0_ = 0.95 mm, *t*_1_ = 0.8 mm, and *t*_2_ = 0.7 mm. To validate the rationality and accuracy of this customized design scheme, physical specimens are fabricated using metal additive manufacturing technology. As shown in Figure 10b, a comparison between the experimental and simulation results demonstrates a high consistency in deformation modes, with both exhibiting the orderly, step-by-step compression characteristic of the ACH-2 structure. Crucially, the maximum error between the experimental results and the target plateau stress requirement is found to be less than 5%, unequivocally verifying the reliability of the proposed customized design method. This study successfully establishes an effective performance customization method for the ACH-2 structure. Though the design method proposed in this study is currently applied only to guiding the performance design of ACH-2 under in-plane loading, the underlying framework possesses theoretical extensibility and can be further adapted to the analysis and design for out-of-plane loading and even high-strain-rate impact conditions.

## 5. Conclusions

In this study, arc-circular lightweight honeycomb structures are designed and analyzed using both experimental and simulation techniques. The main findings are summarized as follows: All configurations exhibit significant multi-stage load responses, with the ACH-2 configuration, which incorporates smaller sub-cells, demonstrating the highest compressive stress and energy absorption potential. Among the three structures, ACH-2 shows the highest energy absorption capacity, followed by ACH-1, with AH exhibiting the lowest performance. Notably, the specific energy absorption (*SEA*) of the ACH-2 structure is enhanced by 210% compared to the baseline AH structure. Within the studied parameter range (wall thickness *t*= 0.2 to 1.0 mm), total energy absorption increases significantly by 29%. This improvement is 5% greater than the increase in the relative density of the structure over the same range, suggesting a positive structural efficiency effect beyond merely adding material. Furthermore, the effect of increasing wall thickness on the two-stage plateau stress is analyzed, with the stress levels of the first and second plateaus increasing by 21% and 36%, respectively. More specifically, the synergistic increase in both *t*_1_ and *t*_0_ simultaneously enhances multiple performance metrics. This combined adjustment results in improvements in total energy absorption, specific energy absorption, and compress stress levels for both the first and second deformation plateaus. While increasing the macroscopic dimension of ACH-2 significantly enhances its energy absorption capacity, the effect of increasing the number of cells on the overall energy absorption performance at the same relative density is relatively limited. Finally, a reverse design framework for ACH-2 with multi-stage plateau stress is successfully established. This study presents an effective performance customization method for the ACH-2 structure, offering a promising approach for the design of structures with multi-stage plateau stress characteristics. This design method can also provide design ideas for passive safety protection of transport equipment, such as traffic and ships, under different collision loads.

## Data Availability

The original contributions presented in this study are included in the article. Further inquiries can be directed to the corresponding author.
